# The role of collagen in cancer: from bench to bedside

**DOI:** 10.1186/s12967-019-2058-1

**Published:** 2019-09-14

**Authors:** Shuaishuai Xu, Huaxiang Xu, Wenquan Wang, Shuo Li, Hao Li, Tianjiao Li, Wuhu Zhang, Xianjun Yu, Liang Liu

**Affiliations:** 1 Department of Pancreatic Surgery, Fudan University Shanghai Cancer Center, 270 Dong An Road, Shanghai, 200032 People’s Republic of China; 2 Department of Oncology, Shanghai Medical College, Fudan University, Shanghai, 200032 People’s Republic of China; 3 Shanghai Pancreatic Cancer Institute, Shanghai, 200032 People’s Republic of China; 4 Pancreatic Cancer Institute, Fudan University, Shanghai, 200032 People’s Republic of China

**Keywords:** Collagen, Cancer, Mutated genes, Signaling pathways, Tumor microenvironment, Prognosis, Resistance, Therapy

## Abstract

Collagen is the major component of the tumor microenvironment and participates in cancer fibrosis. Collagen biosynthesis can be regulated by cancer cells through mutated genes, transcription factors, signaling pathways and receptors; furthermore, collagen can influence tumor cell behavior through integrins, discoidin domain receptors, tyrosine kinase receptors, and some signaling pathways. Exosomes and microRNAs are closely associated with collagen in cancer. Hypoxia, which is common in collagen-rich conditions, intensifies cancer progression, and other substances in the extracellular matrix, such as fibronectin, hyaluronic acid, laminin, and matrix metalloproteinases, interact with collagen to influence cancer cell activity. Macrophages, lymphocytes, and fibroblasts play a role with collagen in cancer immunity and progression. Microscopic changes in collagen content within cancer cells and matrix cells and in other molecules ultimately contribute to the mutual feedback loop that influences prognosis, recurrence, and resistance in cancer. Nanoparticles, nanoplatforms, and nanoenzymes exhibit the expected gratifying properties. The pathophysiological functions of collagen in diverse cancers illustrate the dual roles of collagen and provide promising therapeutic options that can be readily translated from bench to bedside. The emerging understanding of the structural properties and functions of collagen in cancer will guide the development of new strategies for anticancer therapy.

## Background

Cancer continues to receive increasing attention from the academic community because it was the third most common cause of death worldwide in 2018. A total of 18.1 million new cancer cases and 9.6 million cancer deaths were evaluated in 2018 [1], and there are predicted to be 1,762,450 additional cancer cases and 606,880 cancer deaths in the United States in 2019 [2]. Despite various cancer-related guidelines for diagnosis, treatment, and follow-up, improving the long-term prognoses of certain cancer patients remains difficult. Cancer treatment strategies with highly effective response rates still need to be explored. An increasing amount of recent research has concentrated on the function of the tumor microenvironment in favoring cancer progression. In addition, cancer cells exhibit multiple hallmarks of cancer progression, including the recruitment of various cells to form a tumor microenvironment [3], which consists of varying functional stromal cell subtypes and matrix protein polymers [4]. The most abundant matrix protein polymers are collagens, which increase tumor tissue stiffness, regulate tumor immunity, and promote metastasis [5, 6]. In addition, extensive collagen deposition is the main pathological characteristic of some cancers, for which sufficient therapeutic applications are lacking, resulting in the poor survival outcomes of patients [7]. Herein, we summarize the current understanding of the key basic and clinical functions of collagen in cancer and provide clues regarding promising treatments for modifying the tumor matrix.

## Physiological and physicochemical properties of collagen

Collagen is a type of right-handed helix glycoprotein that contains three homologous or nonhomologous left-handed helix α chains. These α chain amino acid sequences are characterized by glycine–X–Y repeats with or without interruptions, with X and Y most likely being proline or hydroxyproline, and the hydroxyproline content of collagen contributes to its thermal stability [8].

Nascent α chains by different genes are encoded first to compose the N-terminus. The next step of assembly into a three-helix structure begins with the C-terminus of the nascent α chains to form procollagen, which is accompanied by certain chaperone proteins including heat shock protein 47, prolyl-hydroxylase, and protein disulfide isomerase to ensure precise alignment [9]. Hydroxylation and glycosylation in the endoplasmic reticulum are two main modifications that occur after translation, and the hydroxylation modification is regulated by vitamin C and pyruvate metabolism [10, 11]. Then, procollagen is hydrolyzed to form collagen by procollagen *N*-proteinase and C-proteinase within Ca^2+^ surrounding the endoplasmic reticulum along with the chaperone heat shock protein 47 and protein disulfide isomerase. This important hydrolysis reaction is the rate-limiting step of collagen biosynthesis. In addition, endopeptidases and metalloproteinases can also excise procollagen at both the N-terminus and C-terminus, and the removed propeptides can conversely regulate the amount of procollagen, further influencing collagen production [12, 13].

Collagen is released into the extracellular matrix (ECM) to form a fibril supramolecular assembly that may start in Golgi-to-membrane carriers after procollagen excision or be localized at the plasma membrane of fibroblasts. The stability of collagen assembly is influenced by intramolecular and intermolecular linkages, particularly covalent linkages, chiefly including lysyl oxidase (LOX) crosslinks [14], glycosylation crosslinks [15], and transglutaminase crosslinks [16], which vary across collagen types.

Different collagens in the ECM are finally degraded by various matrix metalloproteinases (MMPs) belonging to the zinc-dependent endopeptidase family, by proline oxidase, or by sheddases that release the soluble ectodomain of membrane collagens [17, 18].

In general, the 28 known collagen types are classified into four subfamilies on the basis of their supramolecular assemblies, including fibril-forming collagens (I, II, III, V, XI, XXVI, XXVII); fibril-associated collagens with interrupted triple helices (FACITs: IX, XII, XIV, XVI, XIX, XX, XXI, XXII, XXIV), which characteristically link to the surface of collagen fibrils rather than form fibrils by themselves; network-forming collagens (IV, VIII, X), which characteristically generate noncollagenous C-terminal domain dimers and N-terminal 7S domain tetramers; and membrane-anchored collagens (MACITs: XIII, XVII, XXIII, XXV) [19]. Among these types, COLI, COLIII, and COLV are mainly produced by fibroblasts, while COLIV is predominantly expressed by epithelial and endothelial cells. Notably, cancer cells and tumor-associated macrophages also produce collagen under some circumstances [20, 21].

## Cancer cells influence collagen formation

During the occurrence and development of cancer, the ECM undergoes structural changes. In cancer cells, the content and distribution of collagen is modified to further coordinate cancer cell biological properties, including various gene mutations, transcription factors, signal transduction pathways, and receptors.

The heterogeneity of mutated genes is one of the major promoters for cancer cell behavior and influences the interaction between cancer cells and ECM components. The mutation of oncogenes, which are mainly divided into tumor suppressor genes and proto-oncogenes, also alters the collagen conditions in the tumor matrix.

The content and architecture of collagen are strongly altered by mutated tumor suppressor genes in cancer cells. The p53 pathway regulates the formation of tumor-associated collagen signature-3, which is referred to as a collagen bundle angled 60° to 90° relative to the cancer border and is indicated by the proliferation and invasion of cancer [22]. Mutated p53 in cancer cells, along with the activation of Janus kinase 2-signal transducers and activators of transcription (STAT) 3 signaling, influences the collagen production response to paracrine stimulation from pancreatic stellate cells [23]. In addition, the effects of collagen resolvents are associated with p53. The extracellular collagen-derived antiangiogenic factor Arresten, which is located in the C-terminal noncollagenous domain of COL4A1, has been linked to p53 activation [24]. The p53 gene upregulated collagen prolyl-hydroxylase to potentiate the production of full-length COL4A1, further enhancing the content of Arresten [25]. Collagen closely interacts with not only p53 but also other tumor suppressor genes associated with cancer processes. Cancer progression can be regulated by deleting a single copy of the phosphate and tension homology deleted on chromosome ten (PTEN) gene or by completely silencing this gene, resulting in the increased recruitment of cancer-associated fibroblasts (CAFs) and production of COL1A1 [26, 27].

Consistently, mutated proto-oncogenes combine with collagen to support cancer progression. Mutant Kras together with the epithelial-mesenchymal transition (EMT) regulator Snail enhanced collagen production by pancreatic cancer stellate cells, and silencing Kras expression markedly decreased COLI deposition in renal fibrosis [28, 29].

Transcription factors can lead to aberrant target gene expression and tumorigenesis, and nuclear factor kappa-B (NF-κB) and STATs mostly participate in collagen expression and organization. For example, COL2A1 was shown to be under the transcriptional control of the NF-κB subunit p65 in sarcomatous [30]. Collagen fibers showed less parallel alignment, less skewed distribution, and more direction variation rather than decreased numbers following combination treatment with the Janus kinase/STAT3 inhibitor AZD1480 and gemcitabine for pancreatic cancer [31].

Cancer cells further communicate with collagen via signaling pathways during the processes of cellular metabolism, proliferation, differentiation, and apoptosis. Transforming growth factor-β (TGF-β)/Smad signaling is a typical component of serine/threonine kinase signal transduction. Accumulating studies have revealed a positive role of TGF-β/Smad signaling in collagen modification. The architecture and mechanics of collagen fibers adjacent to epithelial lesions, rather than abundant bulk collagen, transformed the pancreatic epithelium into stiff fibrotic tissue via nonfunctional Smad4-phosphorylated myosin light chain 2 [32]. TGF-β sometimes reverses cancer cell functions via collagen. Collagen stiffness induced melanoma differentiation through the Yes-associated protein (YAP)/pax3/microphthalmia-associated transcription factor (MITF) axis, but in the presence of fibroblasts, TGF-β suppressed YAP/pax3/MITF expression and induced YAP/transcriptional enhanced associate domain/Smad-driven transcription, leading to dedifferentiation [33].

Other signaling pathways also affect collagen within cancer cells. Crosstalk between TGF-β and the Ras-Raf-mitogen-activated protein kinase (MEK)-extracellular signal-regulated kinase (ERK) signaling pathway increased collagen synthesis along with p38 activation in melanoma cells to promote cancer progression [34]. Overexpression of tRNA_i_^Met^ increased the production of collagens and collagen-processing enzymes, especially COLII, forming a protumorigenic ECM [35].

Tyrosine kinase receptors are one category of principle collagen-related receptors that are expressed in various cancers. Fibroblast growth factor receptor (FGFR) 4-R388, in which Gly388 in the FGFR4 transmembrane domain was replaced with arginine, regulated the degradation of COLI, COLII, and COLIV by increasing MMP-14 protein expression in prostate cancer cells, especially within the tumor and in the fibrous capsule around the cancer [36]. The effect of epidermal growth factor receptor (EGFR) on collagen remains to be further studied. Collagen was reduced in recurrent breast cancer by combinatorial treatment with FGFR- and EGFR-specific inhibitors, similar to the effect of this treatment on primary tumors [37]. In contrast, c-Met expression, rather than EGFR expression, colocalized with abundant COLI in pulmonary adenocarcinoma [38].

The G protein family receptors, especially small G proteins, including Ras and Rho members, are important in collagen fiber properties and production. G proteins can promote matrix stiffness due to their collagen alignment change. The high stiffness increases nuclear localization of the transcription factor Twist1 by further reducing the expression of the cytoplasmic binding partner Ras-GTPase-activating SH3 domain-binding protein 2 to induce cancer EMT, invasion, and metastasis [39]. Cell division cycle 42, a member of the Rho family, was shown to regulate the thickness and contractility of collagen with the activation of MMP-9 [40]. Rho-associated coiled-coil kinase (ROCK)/Rho signaling may communicate with collagen directly through fibroblasts to regulate cancer cell behavior. At least two interconvertible types of cancer cell migratory motility were shown to be regulated by adhesion to collagen: mesenchymal motility was dependent on integrin and MMPs with Ras-related C3 botulinum toxin substrate 1 (RAC1) signaling and caused cells to appear elongated and bipolar, while amoeboid motility was dependent upon the ROCK/Rho kinase and caused cells to appear round, further leading to myosin-II light chain phosphorylation and actomyosin shrinkage [41]. The ROCK/Rho signaling pathway also influences collagen by other mechanisms. Acetylation of the COL1A1 gene promoter was facilitated by ROCK/Rho signaling pathways in breast cancer cells [42]. In addition, in pancreatic ductal adenocarcinoma (PDAC), collagen impairment via ROCK inhibition was independent of changes in fibroblast proliferation and survival [43]. Notably, the three‐dimensional collagen matrix was remodeled by PDAC cells, possibly by the fusion of ROCK with estrogen receptor (ER) causing increases in MMP-10 and MMP-13 [44].

## The influence of collagen on cancer cell behavior

**Fig. 1:**
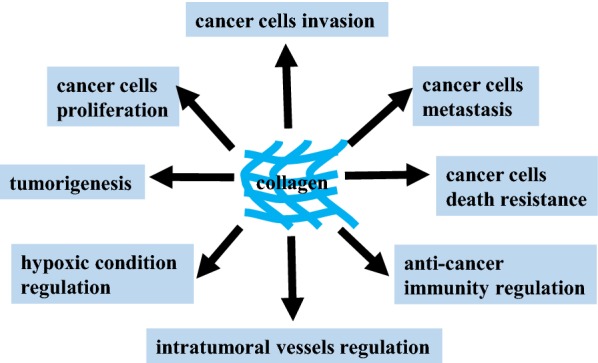
The contribution of collagen to cancer cells

Cellular behavior is controlled by cell signal transduction pathways. Cells accept external signals through receptors and transmit them by cascade, which then transform extracellular signals into intracellular signals, causing physiological cellular reactions that regulate biological activities. Collagen, a component of the ECM, also influences cancer cell behavior (Fig. 1). Cancer cells reversely reshape collagen to form a reinforcing cell-collagen loop, which gradually fosters cancer progression.

Collagen interacts with cancer cells mainly by directly connecting to cancer cell receptors. Discoidin domain receptors (DDRs) are a subfamily of tyrosine kinases that are divided into homologous DDR1 and DDR2 receptors. Collagens closely associate with preferred DDRs, such as COLIV with DDR1 and COLII and COLX with DDR2. A COLIV-DDR1-MMP-9-COLIV feed-forward loop was shown to promote the migration and adhesion of myeloid leukemia cells in bone marrow by activating AKT [45]. DDR1b phosphorylated at Tyr513 by COLI, as opposed to DDR1a, interacted with the signaling adaptor Src homolog 1 to affect focal adhesion kinase (FAK)-related protein-tyrosine kinase, resulting in N-cadherin upregulation in both primary and metastatic PDAC cells to induce EMT [46]. Others further reported that COLI activated DDR2 rather than integrin or TGF-βR to stimulate ERK2 in a Src-dependent manner; activated DDR2 then phosphorylated Snail1 at S82 and S104 and inhibited glycogen synthase kinase (GSK) 3β activity, ultimately contributing to sustained MMP-14 and collagen synthesis in breast cancer [47]. Nevertheless, DDR2 activated by collagen was not conducive to Src homolog 2 domain phosphorylation [48]. Notably, the binding of COL11A1 to both α1β1 integrin and DDR2 to activate the Src-phosphatidylinositol 3-kinase (PI3K)/AKT-NFκB signaling pathways induced the expression of three cisplatin-induced apoptosis inhibitors in ovarian cancer [49].

Adhesion between collagen and cancer cells, such as the adhesion of COLI and COLIV to cancer cells, impacts cancer progression [50]. The cadherin family represents one typical cell adhesion molecule. COLI stimulated E-cadherin upregulation to facilitate the migration of PDAC cells [51]. However, other studies on PDAC have reported the opposite effect of COLI on E-cadherin via different signaling pathways; Smad-interacting protein 1, a member of the small Zfh-1 family that acts as a transcriptional repressor, was induced by COLI to downregulate E-cadherin by simultaneously binding to two defined DNA target sites at E-boxes of the E-cadherin promoter through two zinc-fingers clusters [52]. The COLIV-regulating chemokine (C-C motif) ligand (CCL) 5 and CCL7 were associated with the alteration of E-cadherin to influence EMT, further promoting liver metastasis [53]. COLIV not only promoted a decrease in E-cadherin expression, an increase in N-cadherin expression, and upregulation of Snail1, Snail2, and Sip1 (E-cadherin transcriptional repressors that bind at E-boxes of the E-cadherin promoter) but also induced FAK and ERK1/2 activation in affiliation with TGF-β during EMT, resulting in increased MMP-2 secretion and enhanced cell migration [54]. In addition, the mediation of prostate cancer metastasis by COLXXIII corresponds to changes in OB-cadherin, α-catenin, β-catenin, γ-catenin, vimentin, and galectin-3 protein expression [55].

Integrin, a typical adhesion molecule in cancer cells, often mediates cancer cell behavior, especially when combined with collagen. Integrin comprises two units: α and β. Different types of collagen bind to various integrins in numerous signaling pathways in cancer cells. The binding of integrin to collagen led to the activation of AKT/PI3K signaling, mitogen-activated protein kinase (MAPK) signaling, and Rho family signaling, and the MEK/ERK signaling pathway especially regulated αv integrin subfamily members such as αvβ3 and αvβ5, inducing the proliferation and invasion of squamous cell carcinoma (SCC) cells [56]. Additionally, the deposition of collagen through integrin-regulated ROCK, FAK, and AKT activation inactivated GSK3β and increased the nuclear localization of the mechanotranscription coactivator β-catenin to promote cutaneous SCC progression [57]. Various experiments have further revealed the effects of specific types of collagen in combination with integrin on cancer. COLI is a typical interstitial matrix collagen via integrin to induce cancer cell behavior. Remodeled COLI affected the invasion of ovarian cancer cells by mediating the integrin-PTEN/PI3K/AKT signaling pathway [58]. COLI and α2β1 integrin-promoting cathepsin B-mediated invasiveness were associated with secreted acidic and cysteine-rich proteins in melanoma [59]. The mediation of COLI by α2 integrin led to EMT-like changes, such as downregulated E-cadherin and β-catenin expression, decreased differentiation, increased clonogenicity, and increased colorectal cancer stem cells [60]. The expression of αv integrin response to COLI was enhanced by melanoma cells to promote the upregulation of protein kinase C (PKC) α, thereby relocating endogenous p53 protein [61]. During its adhesion to COLI, mda-9/syntenin at the plasma membrane facilitated processes in the formation of β1 integrin signaling complexes, including the assembly of the integrin-linked kinase (ILK)-PINCH1-α-parvin complex and its translocation to the cell membrane, leading to the activation of AKT, RAC1, and ERK1/2 to promote cancer metastasis [62]. The linearization and matrix compaction of COLI, via β1 integrin-FAK signaling modulated myosin IIA, was exhibited by most radiation-induced breast cancer cells [63]. COLIV accounts for the basement membrane. The high-density COLIV matrix induced the formation of cancer cell invadopodia and actin-rich proteolytic protrusions, which locally degraded collagen via αvβ3 integrin [64, 65]. The COLIV/β1 integrin signaling pathway significantly stimulated Src and ERK phosphorylation, reducing cell stiffness and accelerating cell motility [66]. Ras GTPases, Rac GTPases, PI3K, and PKC participated in melanoma cell migration mediated by COLIV/β1 integrin [67]. In soft-tissue sarcoma, the interplay of COLVI and NG2 triggered PI3K activation through α2β1 integrin, which was associated with adhesion, survival, aggregation, and migration and did not directly influence cell mitosis [68]. Both matrix collagen and basement membrane collagen communicate with integrin to impact cancer cell behavior, and numerous other collagens also bind to integrin to regulate cancer progression. COLXIII in breast cancer [69], COLXVI in glioblastoma [70], and COLXVI in OSCC [71] induce β1 integrin to promote cancer stemness, invasion, and drug resistance. Even collagen glycosylation modulates integrin binding. Galactosylation occurred on the periphery of α2β1 integrin, where it interacted with α1(IV)382–393 but occurred in the middle of α3β1 integrin, where it interacted with α1(IV)531–543 in melanoma cell adhesion [72].

Collagen can stimulate additional signaling pathways in cancer cells to exert various functions. The increased expression of COL1A1 affected the caspase-3/PI3K/AKT pathways to inhibit cell apoptosis in cervical cancer tissues [73]. After the withdrawal of rapamycin treatment, mutated COL1A1 reinforced PI3K–AKT-mammalian target of rapamycin (mTOR) signals in cancer stem cells to sustain the metastatic burden of ERα-positive breast cancer cells; however, lung metastases were independent of mTOR signaling [74]. In addition, increased COLI did not alter primary tumor growth and ERα expression but enhanced circulating cancer cells and metastasizing cancer cells with decreased phospho-STAT5 expression, increased phospho-ERK1/2, and increased phospho-AKT expression; this phenomenon coincided with the formation of invasive protrusions of the primary tumor harboring collagen fibers angled perpendicularly to the tumor mass [75]. However, COLI in non-small cell lung cancer (NSCLC) induced mTOR activation through an AKT-independent pathway, leading to EGFR-tyrosine kinase inhibitor resistance [76]. The Notch3-COL4A2 loop promoted anoikis resistance with a reduction in phosphorylated AKT and ERK 1/2 in ovarian cancer cells [77]. Although both collagen glycation and carbamylation affected the metastasis of cancer cells, glycation caused a more obvious delay in cell adhesion time and deficient actin stress fibers and inhibited the mean cell speed and FAK phosphorylation state more than carbamylation [78]. However, increased collagen in fibrosarcoma tissue inhibited tumor growth and metastasis because the tumor necrosis factor (TNF) receptor 2/p38 MAPK signaling pathway activated collagen expression via gadolinium-containing fullerenol [79]. This distinction implies that different cancer cells facilitate collagen expression to exert inverse effects on cancer progression.

## The relationship among exosomes, microRNAs and collagen in cancer

Recent studies have highlighted the relationship among exosomes, microRNAs (miRNAs) and collagen in cancer [80–82].

MiRNAs are a class of small, noncoding RNAs that act as epigenetic regulators of various physiological and pathological processes [83, 84]. The deregulation of miRNAs is associated with the initiation and progression of many diseases, such as cardiovascular diseases, infectious diseases, diabetes, central nervous system-related diseases, and cancer [85–88]. MiRNAs exert their regulatory activity by affecting a variety of physiological and pathological processes in cancer. Several studies have documented the roles of miRNAs in tumor growth, angiogenesis, and metastasis [89, 90], as well as their utility as diagnostic and therapeutic biomarkers [91–93]. Regarding the influence of miRNAs on collagen, various miRNAs are regulated in cancer cells to affect distinct functions. Breast cancer cells downregulate miR-196b-5p, which decreases COL1A1 levels, to induce growth and metastasis [94]. Intestinal gastric cancer cells suppress COL1A2 via miR-25 to promote EMT and angiogenesis [95]. MiR-27b-3p and miR-455-3p enhance cancer cell quiescence in response to activated p53 to increase drug resistance and recurrence [96]. The miRNA let-7d inhibits pancreatic stellate cell activation [97] and macrophage infiltration by targeting COL3A1 in renal cell carcinoma [98]. Collagen-related enzymes are also differentially regulated by miRNAs in cancer cells. MiRNA-29a targets heat shock protein 47 in cervical SCC [99], and miR-26a/b affects lysyl oxidase-like (LOXL) 2 and procollagen-lysine, 2-oxoglutarate 5-dioxygenase 2 (PLOD2) levels in renal cancer cells [99]. Losartan improves the efficacy of chemotherapy in ovarian cancer partly by inducing antifibrotic miRNAs to normalize the ECM [100].

Exosomes are membrane-enclosed structures that facilitate communication between cancer cells and the ECM to influence cancer cell survival, growth, and metastasis and the immune system [101]. Cancer-derived exosomes induce the formation of CAFs in the collagen matrix to promote EMT [102] and increase the secretion of MMP-14 to regulate collagen [103]. In addition, collagen enhances exosome secretion [104]. Collagen and exosomes form a mutually beneficial feedback loop to promote cancer progression.

## The reciprocity between collagen and cancer cells under hypoxic conditions

Hypoxic conditions, a common condition in collagen-rich ECM, intensify cancer progression based on the interaction between cancer cells and collagen. Hypoxia-inducible factor (HIF)-1, LOX, and MMP participate in the process. COLI fibers are reduced in the hypoxic microenvironment via increased expression of LOX in renal cell carcinoma, decreased expression of MMP-3 in breast cancer, or decreased expression of MMP-1 and MMP-16 in prostate cancer [105, 106]. Collagen can be remodeled by HIF-1 regulating LOXL1, LOXL2, and LOXL4 [107].

Other substances, such as hepatitis transactivator protein X in liver cancer [108], LKB1 via COLIV and β1 integrin in lung cancer [109], and HIF1α-dependent PLOD2 in primary sarcomas and pulmonary metastasis [110], participate with collagen in the HIF/LOX pathway. Hypoxia increased the expression of procollagen-lysine as collagen crosslinker to further enhance collagen fiber size and thus promote cancer metastasis [111]. COLI fibers exhibited covalent crosslinking with prolyl 4-hydroxylase alpha 1 and prolyl 4-hydroxylase alpha 2 in differentiated cell types of triple-negative breast cancer, and the prolyl 4-hydroxylase alpha 1/HIF-1 axis increased chemoresistance [112, 113]. Even mutant p53 regulated the expression of COL7A1 in NSCLC, not by influencing HIF-1 binding to DNA, but rather by inhibiting its transcriptional activity [114].

Hypoxic conditions are closely associated with vessels in cancer. Matrix collagen stiffness, rather than collagen density, alters vascular growth and integrity. Moreover, increased fibril density decreased vessel network formation, while increased interfibril branching improved vessel volume density and formation, which were markedly dependent on the changed temporal and spatial depositions of COLIV [115]. In addition, neovessel branching is associated primarily with collagen crosslinking rather than with collagen content [116]. Notably, the von Hippel–Lindau protein directly regulated COL4A2 assembly independent of HIF-α to restrain cancer angiogenic formation [117]. Further reflecting the relationship between collagen and cancer cells with regard to treatment, improved tumor stromal pO2 levels and the recovery of blood flow were associated with the content and diameter of collagen fibril under imatinib treatment [118].

## Interaction between collagen and tumor matrix components

**Fig. 2:**
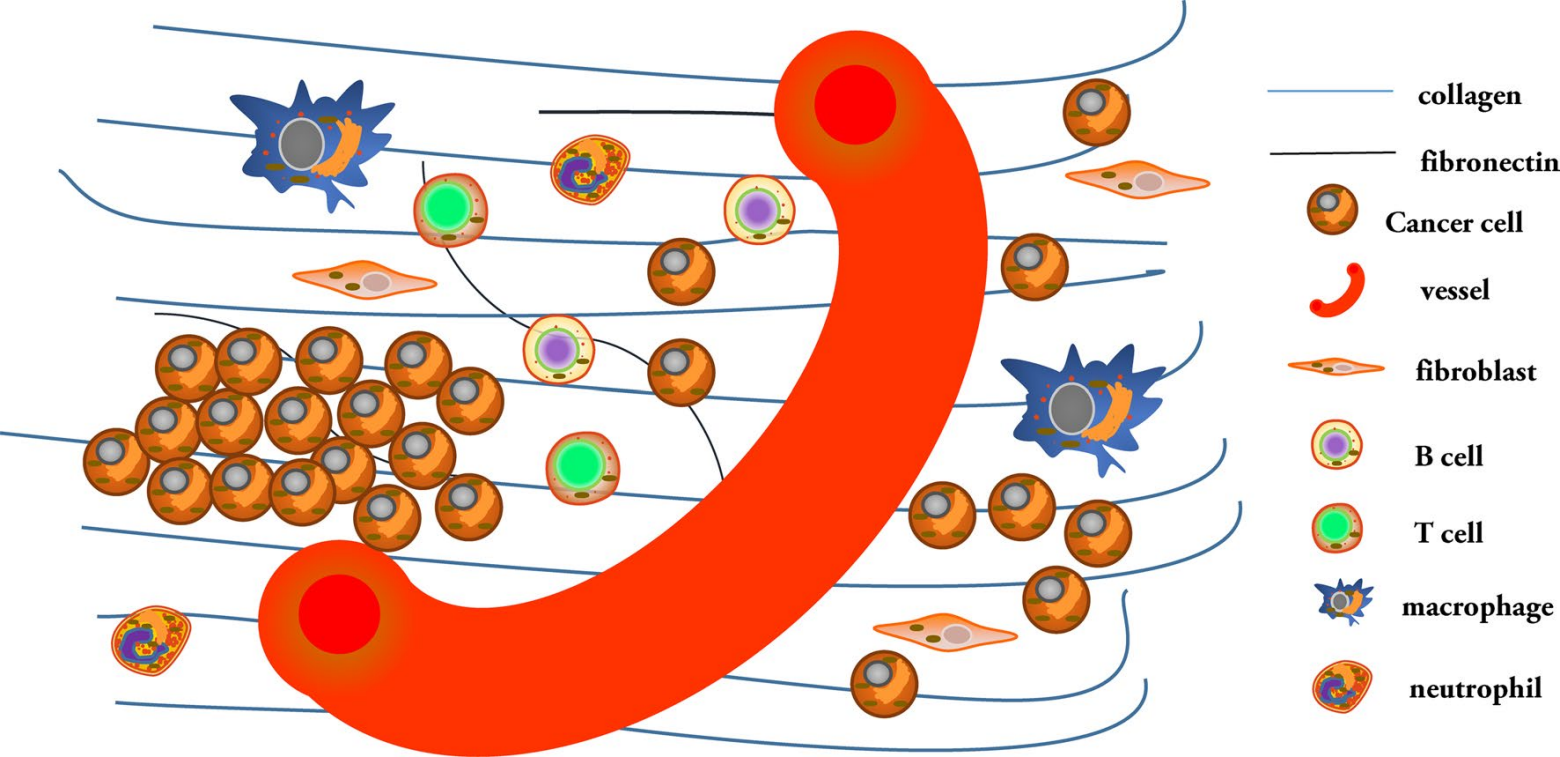
The complexity between collagen and the extracellular matrix. Multiple stromal cells and the extracellular matrix play important roles in stimulating or inhibiting collagen functions via different pathways in cancer progression. Collagen-rich extracellular matrices can bind to other molecules to form dense fibrosis, which induces an anoxic environment and changes the condition of new blood vessels. The behavior of cancer cells is also closely related to collagen. This process also affects the activity and localization of innate and adaptive immune cells

The complex interactions among collagen and matrix proteins within ECM cells contribute to cancer initiation and progression, as shown in Fig. 2.

Collagen in cancer tissue accompanies various categories and degrees of myeloid-lineage immune cells, including mast cells, macrophages, and neutrophils, and lymphocytes, including T cells and B cells, to reflect different cancer progression stages [119]. For example, pancreatic cancer stromal compositions were classified as inert, dormant, fibrogenic, or fibrotic based on α-smooth muscle actin (SMA) and COLI expression; these differing stromal compositions individually influenced the levels of CD4^+^ T cells, CD8^+^ T cells, macrophages, and neutrophils [120]. Increased infiltration of macrophages and lymphocytes was also observed in subcutaneous adipose collagen in gastrointestinal cancer patients with cachexia [121]. However, increased stromal COL10A1 was accompanied by low numbers of total tumor-infiltrating lymphocytes in ER-positive/EGFR2-positive breast cancer [122].

The location and quantity of collagen with lymphocytes reinforce discrepant cancer progression via distinct signaling pathways. A high-density collagen matrix, rather than a low-density matrix, decreases T cell abundance and cytotoxic T cells but upregulates regulatory T cells [123, 124]. Aligned fibers and collagen density around vascular regions and epithelial cancer cells restricted the migration of T cells and limited their entry into the tumor mass [125]. Moreover, others have further reported that the distribution of collagen with lymphocytes affects cancer progression, but the effects on different cancers remain distinct. COLI was not associated with T cell deficiency in PDAC [126]. However, others noted that the metastatic urothelial cancer response to programmed death-1 and programmed death-ligand 1 treatment involved the movement of CD8^+^ T cells from the cancer parenchyma to the collagen-rich peritumoral stroma [127]. Nevertheless, contact between infiltrating T cells, Tregs and antitumor T cells with collagen was inhibited by members of the TGF-β pathway to provide antimelanoma-immunity [128, 129].

Furthermore, both adaptive immunity and innate immunity affect collagen. Collagen deposition and linearization are positively related to macrophage activity [130]. Additionally, denatured collagen acts as a strong chemoattractant for macrophages that mediate the promotion of cancer [131]. The possible reasons for these functions of collagen are described below. Macrophages closely interact with stellate cells to influence the collagen matrix [132]. Tumor-associated macrophages orchestrate the deposition, crosslinking, and linearization of collagen fibers, specifically COLI, VI, and XIV, at areas of tumor invasiveness [133]. Although macrophages do not exhibit efficient collagen internalization of mesenchymal origin, those originating from circulating CCR2 monocytes internalize collagen in an MMP-dependent manner, which is mediated by the mannose receptor rather than by β1 integrin [134–136]. In the collagen-rich breast cancer environment, coexpression of fibroblast activation protein and heme oxygenase-1 in macrophages resulted from an early innate regenerative response to IL-6 to directly facilitate transendothelial migration [137]. Cyclooxygenase-2 also induced overall collagen deposition and macrophages in early-stage breast cancer [138]. Notably, in the context of an αvβ3 integrin-specific collagen hydrogel, M2-like immunosuppressive macrophages promoted angiogenesis in glioblastoma, while M1-like proinflammatory macrophages suppressed angiogenesis, which was regulated by Src-PI3K-YAP signaling associated with TGF-β1 [139]. IL-10 induces M2 differentiation in heterospheroid macrophages [140]. In contrast, collagen degradation is enhanced not by M2 macrophages but rather by M1 macrophages, as endogenous macrophages are recruited, modified and activated to produce MMPs and hepatic growth factors, thereby enhancing hepatocyte proliferation and the release of TNF-related apoptosis-inducing ligands by natural killer cells, leading to hepatic stellate cell apoptosis [141]. Moreover, there are other important innate immune cells in the ECM that exhibit anticancer immunity or immunosuppressive effects. Collagen deposition can be induced by neurofibromatosis type 1-recruited mast cells, which mediate stem cell factor/c-kit signaling in neurofibromas [142]. Collagen-dense tumors increased granulocyte monocyte-colony stimulating factor, which was associated with neutrophil signaling [143]. However, COLXVII overexpression in cancer cells by activated leukocyte-associated immunoglobulin-like receptor-1 diminished natural killer cell cytotoxic activity [144]. By interaction with collagen in ovarian and breast cancer, tumor-associated dendritic cells expressed the highly receptor CD305/leukocyte-associated immunoglobulin-like receptor-1 [145].

Fibroblasts are another important cell type in the ECM. CAFs cause various collagen deposits to develop into intratumoral fibrosis, resulting in cancer occurrence, differentiation, and invasion [4]. These CAFs can even originate from adipose tissue-derived stromal cells [146]. CAFs are classified as tumor-restraining, tumor-promoting, secretory, or ECM-remodeling cells [147], which also exhibit CAF functions via mutated genes [148], the secreted cytokines IL-1β, TNF-α and NF-κB, inflammatory signals, epigenetic regulation, etc. [6]. Cancer cells can invade collagen with fibroblasts, which are characterized by cell cycle phases and small cancer cell nest formation [149]. Additionally, CAFs exert anticancer and cancer-induced effects to show dual influences for cancer progression [150].

Some ECM proteins, mainly including MMPs, hyaluronic acid, fibronectin, and laminin, regulate cancer cell invasion and migration with collagen. The functions of MMPs with collagen are summarized in Table 1, and MMP activity with contractility necessarily establish the ECM stiffness associated with collagen bulk and distribution [151]. The collagen gel storage modulus with glycosaminoglycans chondroitin sulfate and hyaluronic acid was dependent on both the fiber diameter and network mesh size [152]. When cancer cells encountered stiff collagen fibers at fibronectin-rich invasive fronts, they engaged αvβ1 integrin to recruit vinculin and zyxin to focal adhesions sites in a tension-dependent manner to induce PI3K signaling [153]. Similarly, with increased COLI in the laminin-rich ECM regarded as an early tumor microenvironment, cancer stem cells maintained their endothelial-like gene signatures and secreted high levels of VEGFR-2 in a paracrine and autocrine manner to simulate progression [154]. In contrast, unlike the laminin-rich microenvironment, a COLI Matrigel was sufficient to induce colon cancer mesenchymal gene expression, suppressing hepatocyte nuclear factor 4α and its target genes [155]. Additionally, CAFs initially constructed the ECM network by depositing fibronectin, followed by the preferential interaction of COLI with relaxed fibronectin, which, in turn, limited the stretching and mechanical unfolding of fibronectin, leading to collagen superseding fibronectin [156, 157]. Other types of collagen also have functions. The expression of COLIV was associated with the expression of fibronectin and laminin during central nervous system metastasis [158]. The binding of COLXV, rather than I, III, IV, and V, to fibronectin, laminin, and vitronectin inhibited the adhesion and migration of fibrosarcoma cells [159].

**Table 1:** Pathological functions of MMPs associated with collagen in cancer

Subtype of MMPs	Associated collagen	Pathological functions of collagen	References
MMP-1	COLI	Regulated to facilitate melanoma cell growth and invasion	[227, 228]
	COLIV	Regulated to foster breast cancer cell invasion in response to prolactin	[229]
MMP-2	COLI	Modulating MMP-2 activation in osteosarcoma	[230]
	COL4A2	Modulating MMP-2 activation and activity in liver cancer	[231]
	COLI and COLIV	Regulated by the knockdown of MMP-2 to induce cancer metastases	[232, 233]
	Collagen organization	Regulated to enhance malignant glioma recurrence and resistance to vemurafenib	[234, 235]
MMP-3	COLI	Regulated to induce mammary epithelial cells invasion and morphogenesis with chaperone heat-shock protein 90	[236]
	COL11A1	Regulated to promote ovarian cancer progression	[237]
MMP-7	COLI	Both to predict the prognosis of opisthorchiasis-associated cholangiocarcinoma	[238]
MMP-9	COLI	Degraded in invasive melanoma fronts	[239]
	COLI	Potentiated in *Opisthorchis viverrini*-induced cholangiocarcinogenesis	[240]
	COLIV	Degraded to facilitate venous invasion in PDAC	[241]
MMP-10	COLI	Regulated by TGF-β in keratinocytes to promote invasion	[242]
MMP-14	COLI	Increased to promote fibrosis by TGF-β signaling in PDAC	[243]
	COLI	Modulating MMP-2 and MMP-14 activation via β1 integrin	[244]
	COLI	Regulated to prevent apoptosis to promote luminal-like breast cancer progression	[245]
	COLI	Sustained activation of MMP-14 with EGFR at the cell surface enhances invasion, whereas growth within three-dimensional collagen is inhibited	[246]
MMP-16	COLI	Supported around melanoma cells to enhance lymphatic invasion	[247]
MMP-28	COLII	interacted to more adhesion and less migratory	[248]

## Collagen and clinical applications

Various experiments and clinical data have revealed collagen to be a prognostic factor correlated with cancer differentiation, cancer invasion, lymph node metastasis, and clinical stage in cancer patients (Table 2). Lower COLI and COLIV content is a biomarker of differentiated tumors and proliferation potency of cancer cells [160, 161]. The increased expression of COLIV and COLVI and the collagen structure reflect tumor angiogenesis and glioblastoma progression [162]. Even hypomethylation of the COL17A1 promoter is associated with advanced stage, increased invasion of breast cancer, lung adenocarcinoma, cervical cancer, neck SCC, and lung SCC [163].

**Table 2:** Collagen as a prognostic factor for cancer patients

Subtype	Condition	Cancer	Associated clinical significance	References
COLI	Intactness	Colorectal cancer	Changes dynamically at stages I to IV, peaking at stage II	[249]
	Intactness	Prostate cancer	Metastasis	[250]
	COL1A1	Breast cancer	Development and progression along with COL3A1 and COL4A1	[251]
	COL1A2	Colorectal cancer	Proliferation, migration, and invasion	[252]
	COL1A2	Hepatocellular cancer	Metastasis	[253]
	N-terminal telopeptide	NSCLC	Overall survival	[254]
	N-terminal telopeptide	Head and neck SCC	Overall survival along with N-terminal telopeptide of COLIII	[255]
	Pyridinoline crosslinked C-terminal telopeptide (serum)	Breast cancer	Recurrence	[256]
	N-terminal telopeptide (urine)	Breast cancer with bone metastases	Survival prognosis with zoledronic acid treatment for 3 months	[257]
COLII	COL2A1	High-grade serous ovarian cancer	Recurrence	[258]
	COL2A1	Chondrosarcoma	Frequent mutations	[259]
COLIII	COL3A1	Breast cancer	Irregular margin status and mitotic activity	[260]
COLIV	Intactness	Advanced gastric carcinoma	The depth of wall penetration and stage	[261]
	Intactness	Oral SCC	Positive lymph node status	[262]
	Intactness	Colorectal cancer	Liver metastases	[263]
	7S domain (serum)	Hepatocellular carcinoma	Intractable ascites	[264]
	COL4A1	PDAC	Aggressive progression	[265]
	COL4A1 (urine)	Bladder cancer	Recurrence	[266]
	COL4A3	Gastric carcinomas	Cancer size, lymphatic invasion, venous invasion, TNM stage, and histologically distinction	[267]
COLV	Intactness	Resected NSCLC	Overall survival	[268].
COLVI	COL6A1	Cervical cancer	Overall and recurrent-free survival	[269]
COLXI	COL11A1	Esophageal SCC	Advanced clinical stage and lymph node metastases	[270]
COLXIII	COL13A1 (urine)	Bladder cancer	Recurrence	[266]
COLXVII	Intactness	Colorectal cancer	Invasion and metastasis	[271]
	Intactness	SCC	Invasion	[272]
	Intactness	Colon cancer	Metastasis	[273]
COLXXIII	Intactness (tissue and urine)	NSCLC	Recurrence	[274]

Factors other than collagen content are correlated with clinical outcome [164], such as collagen alignment and distribution, which also affect cancer. During tumor progression, collagen exhibits different signatures. Tumor-associated collagen signature 1 (TACS-1) indicates the presence of dense collagen near the cancer, TACS-2 represents collagen fibers parallel to the tumor edge, and TACS-3 depicts radially aligned collagen fibers [165]. TACS-3 was related to cancer cell invasion and poor survival in breast carcinoma and in situ breast ductal carcinoma [166, 167]. TACS-3 was shown to be driven by increased plasminogen activator inhibitor 1 via ERK signaling and promoted the migration of triple-negative breast cancer cells [168]. The elevated density and depth of collagen deposition showed high proliferation and invasion of cancer cells [169, 170]. Although COLI and COLIV are expressed in different tumor stroma compartments in pancreatic cancer tissue, they stimulate proliferation, migration, and antiapoptosis. The main form of COLI was generated by pancreatic stellate cells attributed to cancer cells in an indirect contactable desmoplastic area to activate TGF-β, while COLIV was produced by cancer cells themselves to form an autocrine loop in direct proximity to cancer cells, causing discontinuous basement membrane-like structures that interacted with the COLIV CB3 region and β1 integrin of the cancer cells [171]. However, another study showed that COLV was expressed by pancreatic stellate cells via paracrine loops in PDAC [172]. The desmoplastic reactions in primary cancer were divided into mature, intermediate, and immature based on the presence of keloid-like collagen and myxoid stroma; immature desmoplastic reactions were associated with higher T and N stages, more extensive liver metastasis, and higher recurrence rate than other reaction types [173]. Furthermore, COLVII positively regulates the abundance of the cell polarity markers E-cadherin and B-catenin [174]. The mechanisms underlying these important collagen properties are described below. Among five collagen parameters (alignment, density, width, length, and straightness), increased collagen width is the most powerful parameter for predicting cancer prognosis [175]. The elasticity of the collagen matrix is controlled by fibril bending stiffness rather than by fibril diameter or intrafibrillar crosslinking [176]. Increased collagen fiber alignment, elevated levels of immunoreactive glycosaminoglycans such as heparan sulfate and chondroitin sulfate, and decreased levels of the proteoglycan decorin enhance the stiffness of carcinoma tissues [177]. In contrast, others have notably reported that the distribution of collagen had no effect in the regenerated cervical tissue following excisional cervical intraepithelial neoplasia [178].

Collagen plays an important role in therapy resistance. In esophageal SCC, increased collagen content was associated with chemotherapy resistance via the MAPK and PI3K/AKT pathways [179]. Even at metastatic sites, collagen crosslinking increases tissue stiffness to promote resistance to treatment [180]. Furthermore, increased collagen content was accompanied by increased hyaluronan accumulation, contributing to doxorubicin drug resistance in pancreatic cancer [181]. Specifically, different collagen types exhibit distinct treatment resistances. COLI induced resistance to drugs, such as cisplatin and mitoxantrone, by activating β1 integrin followed by the FAK/PI3K/AKT pathway in ER-positive cancer cells, the MAPK pathway in triple-negative cancer cells, the coexpression of LOX with COL1A2 in ovarian cancer, and the TGF-β1/Smad3-mediated expression of COLI and COLIII in bromocriptine-resistant prolactinoma cells [182–184]. Other mechanisms of drug resistance include COLI-induced tau upregulation, resulting in paclitaxel resistance in ovarian carcinoma [185]. The resistance to radiation of renal cancer cells with intact COLI rather than micronized COLI was mediated by apoptosis attenuation rather than cell cycle redistribution via the PI3K/AKT pathway [186]. In addition, other collagens also play important roles in therapy resistance. COL3A1 optimally predicted the absence of a response to neoadjuvant treatment in rectal cancer and the resistance of ovarian cancer cells to topotecan and paclitaxel [187, 188]. High COLVI expression promoted anoikis resistance and affected the response of salivary gland cancer to radiotherapy and colorectal cancer to adjuvant chemotherapy [189–191]. The reactions of cancers to antiangiogenic therapy have also been closely associated with COLIV. The reduced binding of collagen to PDAC cell surface receptors promoted resistance to VEGF therapy via TGF-β signaling [192], but antiangiogenic therapy increased intratumoral adenovirus distribution by decreasing COLIV [193]. Another study showed that the correlation of bevacizumab with elevated tumor stiffness was driven by hypoxia, leading to increased hyaluronic acid and sulfated glycosaminoglycan contents without significantly changing collagen deposition [194]. COL11A1 induced chemoresistance and exerted antiapoptosis effects in ovarian cancer cells by mediating the transcriptional activation of NF-κB to upregulate the Twist family [195].

Collagen can also be used for the imaging of targeted sites. Imaging of the collagen content and arrangement is conducive to the assessment of tissue stiffness, metabolism, and drug resistance [196–198], and technology to utilize these data is currently under development [199]; for instance, magnetic resonance apparent diffusion coefficient values were used to indicate a negative correlation of collagen with esophageal SCC cells [200]. The label-free Raman spectroscopic measurements used to distinguish radiation-sensitive tumors were based on differences in collagen content [201].

**Fig. 3:**
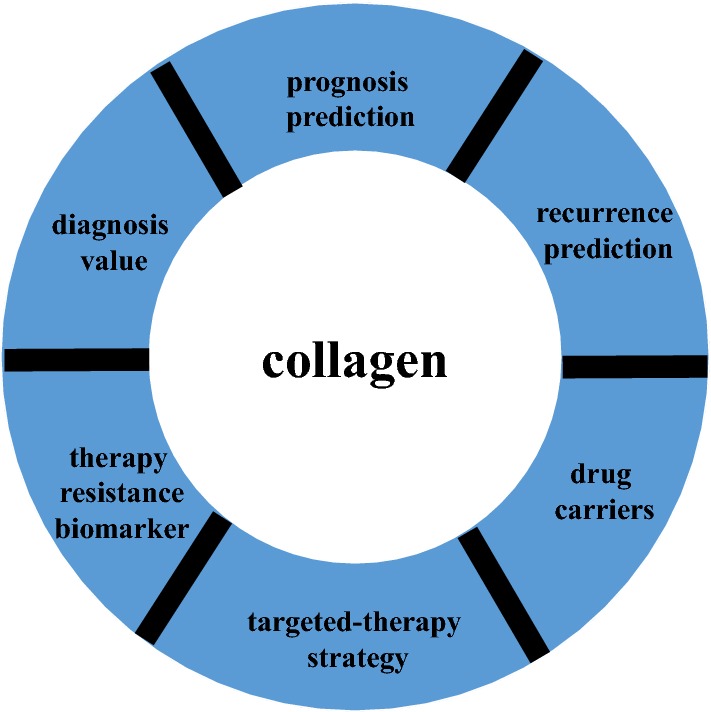
The value of collagen in clinical applications

Because collagen is closely associated with clinical outcome, it can also be used in clinical applications (Fig. 3). Collagen can be utilized as a predictor of prognosis and recurrence, in diagnosis, as a therapy resistance biomarker, as a targeted-therapy strategy, and as a drug carrier.

## Collagen-related therapy

Cancer resistance has substantially hindered the ability to control cancer. Therefore, both cancer cells and tumor microenvironment must be treated, and collagen is a potential target. Moreover, collagen has obvious genetic stability, and its spatial structure remains relatively stable. As collagen is basically present in various types of cancer, the treatment value of modifying collagen conditions in cancer is worth exploring.

Collagen can be regulated by different types of inhibitors against biosynthesized processes and distribution arrangements by interfering with collagen biosynthesis enzymes, disturbing cancer cell signaling pathways, mediating ECM components, or directly utilizing collagenases (Table 3). Although many types of inhibitors exist, most have been investigated in only cell and animal experiments. These indirect methods of modifying collagen are also partially attributed to other intricate related molecular mechanisms and signaling pathways. The treatment of collagen in the current study is mainly to target the function of CAFs. While quiescent CAFs are characterized by the presence of lipid droplets loaded with vitamin A in the cytoplasm, these perinuclear lipid droplets disappear and express the activation marker α-SMA after CAF activation [202]. Moreover, low expression of retinoid receptors is associated with poor survival of pancreatic cancer [203]. Therefore, the vitamin A analog all-trans retinoic acid in the treatment of PDAC induces CAF to be at quiescent status and inhibits cancer cell migration and EMT; however, the relevant clinical trials are only stage I in the recruitment state (NCT03307148) [204]. In addition, some studies focus on the interaction of cancer cells with collagen, which regulates collagen by targeting cancer cells. For instance, the development of PDAC is also associated with vitamin D, which affects insulin synthesis and secretion via reduced CYP24A1 in islets but impairs the anti-proliferation in transformed duct cells via increased CYP24A1 [205]. The vitamin D signaling pathway has the functions of anti-proliferation, pro-differentiation, anti-inflammation, pro-apoptosis, and immune regulation. There is also a stage III clinical trial recruiting to study the influence of high-dose vitamin D3 intake on pancreatic cancer surgery outcomes (NCT03472833). Moreover, vitamin D receptor ligand calcipotriol regulates CAFs to reprise the quiescent state [206], and paricalcitol was selected to participate in ongoing clinical trials on PDAC therapy (NCT03883919, NCT03415854). Hydroxychloroquine can also effectively inhibit the proliferation and metabolic activity of fibroblasts for cancerous interstitial fibrosis and inhibit cancer cells autophagy. However, the combination of gemcitabine hydrochloride and nab-paclitaxel plus hydroxychloroquine did not increase overall survival for metastatic pancreatic cancer; thus, the clinical effect still needs to be further explored [207]. Notably, although MMPs degrade collagen, they also induce cancer angiogenesis; thus, they have complex and paradoxical effects on cancer progression. The combination of an anti-MMP-9 antibody and nab-paclitaxel-based standard cytotoxic therapy was shown to decrease COLI and the metastatic burden compared with that achieved with nab-paclitaxel-based standard cytotoxic therapy in PDAC mouse models [208]. Moreover, collagen-targeted treatments have paradoxical effects on drug delivery and treatment efficacy within the same cancer type. In particular, collagenases monotherapy for cancer may have obvious side effects, and it may even exert opposite effects than those intended. Addition of the sonic hedgehog antagonist vismodegib to gemcitabine did not improve the survival outcomes of metastatic pancreatic cancer patients in a phase Ib/II trial [209]; in contrast, halofuginone disrupted collagen barriers to effectively deliver the drug and promote anticancer immunity [210]. While the antifibrotic drug pirfenidone was effective in early-stage liver fibrosis, it did not influence advanced liver fibrosis and initiation-promotion liver cancer [211].

**Table 3:** Typical inhibitors and drugs that regulate collagen biosynthesized processes and collagen distribution arrangement in cancer studies

Effects of inhibitors	Targeted sites of inhibitors	Typical inhibitors and drugs	References
Interfering collagen biosynthesis enzymes	Collagen genes	MiR-129-5p, MiR-29b, MiR-384	[275–277]
	Prolyl 4-hydroxylase	Budesonide, catechol, *N*-oxalylglycine, coumalic acid, ethyl dihydroxybenzoic acid	[278–280]
	Heat shock protein 90	1G6-D7, dipalmitoyl-radicicol, 17-DMAG, ganetespib	[281–284]
	Heat shock protein 47	MiR-29, 1,3-dimethylol-5-FU, AK778, pirfenidone, terutroban	[285, 286]
	Matrix metalloproteinases	Gallium complex GS2, isoflavonoids, bisphosphonates	[287, 288]
	Lysyl oxidases	Beta-aminopropionitrile	[289]
Disturbing cancer cell signaling pathways	Snail transcription factors	Toosendanin, ponicidin, ferulic acid	[290]
	Hypoxia‐inducible factor	Tamoxifen, 28-*O*-propynoylbetulin	[291, 292]
	STAT3 signaling pathway	VS-4718, stattic, ruxolitinib, S3I-201	[293, 294]
	TGF‐β signaling pathway	LY2157299 monohydrate, trabedersen, fresolimumab, galunisertib	[295, 296]
	NF‐κB signaling pathway	Honokiol, aspirin, ormeloxifene	[297–299]
	AKT signaling pathway	Quetiapine, pirfenidone	[300]
	Notch signaling pathway	Rovalpituzumab tesirine, taladegib, crenigacestat, MiR-148a	[301]
	Hedgehog signaling pathway	Itraconazole, sonidegib, vismodegib	[302]
	RAS signaling pathway	Perindopril, losartan	[100, 303]
	Tyrosine kinase receptor	Bevacizumab, imatinib, ponatinib, dasatinib	[304, 305]
	Discoidin domain receptor	WRG-28, 7rh, AZD0156	[306–308]
	G protein family receptor	AT13148, KD025, Azaindole 1, chelerythrine	[309]
	Integrin	Cilengitide, volociximab, intetumumab, LM609	[310]
Mediating tumor matrix components	Macrophage	Bone-marrow-derived macrophages infusion	[141]
	T cell	Tumor-targeted trimeric 4-1BB-agonistic antibody	[311]
	Cancer-associated fibroblasts	ABT-199, 5-AZA, ismodegib, metformin, Nab-paclitaxel	[312, 313]
	Hyaluronic acid	Halofuginone	[314]
Directly utilizing collagenase	Collagen antibody	Collagen-binding EGFR single-chain Fv antibody fragment	[224]
	Nanoparticle	Poly-lactic-*co*-glycolic acid nanoparticle, Lipid-bilayer mesoporous silica nanoformulations	[222, 315]
	Oncolytic adenovirus	oH(E)mT-DCN, LOAd703	[316, 317]

Combining collagen inhibitors and standard therapeutics, such as chemotherapy and radiotherapy, is a promising anticancer strategy. Collagenase combined with trastuzumab via thermosensitive hydrogels exerted an anticancer effect on animals [212]. Nitric oxide activated endogenous MMP-1 and MMP-2 to deplete collagen, and it significantly improved anticancer efficacy while exerting no overt toxicity in animal models [213]. Under hypoxic cancer conditions, hyperbaric oxygen therapy decreased collagen deposition to enhance chemotherapy efficacy and photodynamic upconversion nanophotosensitizer cancer therapy [214].

Preclinical studies on collagen-related therapy partly demonstrated encouraging outcomes. However, few clinical trials have been conducted, and most have focused on signaling pathways or receptors. These indirect collagen-related treatments remain controversial due to the potential effects of other mechanisms. For example, cilengitide inhibiting αvβ3 and αvβ5 integrins to influence the connection with cancer cells and collagen did not show evident clinical benefits [215]. There are major drugs of direct collagen depletion or collagen alignment changes, but their effects are not clear and need to be further explored (Table 4). Confusing and conflicting results for anti-collagen therapy suggest complex collagen properties. As revealed herein, physiological, pathological and clinical analyses reveal the dual roles of collagen. Collagen has essential functions in normal tissues but also plays important roles in cancer progression and clinical outcomes. Collagen depletion results in the activation of residual cancer cells and incomplete ECM and microvessels. Collagen can function as a barrier for certain stages of cancer, but it can also enhance other stages of cancer. In addition, although collagen can be degraded, its decomposition products can continue to function and thus promote cancer angiogenesis and invasion. The interaction of collagen with other components in the ECM also shows dual effects on cancers. CAFs, which mainly produce stromal collagen, express anti-tumor and tumor-promoting effects; epithelial and endothelial cells, which mainly produce the collagen of basement membrane, can not only maintain vascular stability but also promote cancer angiogenesis and contribute to cancer cells penetration into blood vessels. The individual differences, genetic heterogeneity, and epigenetic heterogeneity of cancer patients can also affect the efficacy of drugs in clinical trials. Therefore, balancing the content, crosslinking, alignment, and distribution of collagen may be a reasonable strategy for cancer treatment. Nanoparticles, nanoplatforms, and nanoenzymes exhibit the expected gratifying properties.

**Table 4:** Collagen-targeted agents directly influencing collagen content and distribution in clinical trials for cancers

Drug	Combination drugs	Cancer	Phase	Status	Result	Reference number
LDE225	Gemcitabine, Nab-paclitaxel	Locally Advanced or Metastasized Pancreatic Cancer	I/II	Unknown		NCT02358161
EN3835	None	Uterine Leiomyoma	I	Completed	Enough safety and efficacy, and decreased tumor bulk	NCT02889848
EN3835	None	Lipoma	II	Completed	dose escalation study	NCT01613313
Losartan	Proton beam radiation, FOLFIRINOX	Locally Advanced Pancreatic Cancer	II	Active, not recruiting		NCT01821729
TRC093	None	Locally Advanced or Metastatic Solid Tumors	I	Completed	Dose escalation study	NCT00492830
Halofuginone hydrobromide	None	Human immunodeficiency virus-related Kaposi’s sarcoma	II	Completed	No clear clinical benefits	NCT00064142

Nanoparticles influence the use of collagen for anticancer therapy in animal models, including combinations with other chemotherapy drugs and treatment regimens [216]. Losartan nanoparticles significantly decrease collagen content to improve tumor penetration and tumor treatment efficiency [217], and losartan in combination with photodynamic nanoplatforms suppressed tumor volume in a breast cancer mouse model [218]. The implantation of nanoparticle/losartan-loaded hydrogel enhanced the intratumoral distribution and anticancer effect of nanoparticles in mice [219]. In addition, supplementation with other nanoparticles further orchestrated collagen normalization rather than destruction, thereby improving the survival rates of cancer patients [220]. A near-infrared light irradiation-activated semiconducting polymer nanoenzyme efficiently digested collagen, leading to nanoparticle accumulation in cancer tissue and the consequential improvement of photothermal therapy [221, 222].

Collagen can also act as a drug carrier or a drug targeted site. Hybrid collagen-cell penetrating peptide carriers improved resistance to enzymatic degradation [223]. Treating cancer with the single-chain fragment of cetuximab along with its collagen-binding domain demonstrated effective results [224]. Collagen affinity can also be used to mediate targeted immunotherapy antibodies. Fusion of the collagen-binding protein lumican and cytokines increased the efficacy of systemic immunotherapies in a melanoma model [223]. The therapeutic use of immune checkpoint inhibitors and interleukin-2 by conjugation (for antibodies) or recombinant fusion (for cytokine) to the von Willebrand factor A3 domain (a collagen-binding domain) eradicated tumors and exhibited obvious safety and efficacy in a breast cancer model [225]. Cancer-collagen-targeting immunoconjugate therapy was evidently applicable to anticancer therapy [226].

## Conclusions

Cells and molecules in the tumor microenvironment have dual effects on cancer progression. The role of collagen is a double-edged sword in cancer. On the one hand, collagen, cancer cells, other cells, and other matrix molecules mutually form an inter-reinforcing loop. This loop contributes to the development of cancer by inducing cancer cells proliferation, migration, and metastasis. On the other hand, preclinical and clinical studies have demonstrated that collagen may slow the development of cancer cells to some extent under some conditions. In summary, the association of collagen with cancer is only partially understood, and future studies are needed to elucidate detailed collagen biological mechanisms in cancer tissue that can be applied to precisely regulate collagen balance to achieve the maximum benefit of treatment. This new strategy combined with other treatment modalities can ultimately improve patient survival and quality of life.

## Data Availability

Not applicable

## Abbreviations

CAFs: cancer-associated fibroblasts
CCL: chemokine (C-C motif) ligand
COL: collagen type
DDR: discoidin domain receptor
ECM: extracellular matrix
EGFR: epidermal growth factor receptor
EMT: epithelial–mesenchymal transition
ER: estrogen receptor
ERK: extracellular signal-regulated kinase
FACITs: fibril-associated collagens with interrupted triple helices
FAK: focal adhesion kinase
FGFR: fibroblast growth factor receptor
GSK: glycogen synthase kinase
HIF: hypoxia-inducible factor
LOX: lysyl oxidase
LOXL: lysyl oxidase-like
MAPK: mitogen-activated protein kinase
MEK: mitogen-activated protein kinase
MITF: microphthalmia-associated transcription factor
miRNA: microRNA
mTOR: mammalian target of rapamycin
MMP: matrix metalloproteinase
NF-κB: nuclear factor kappa-B
NSCLC: non-small cell lung cancer
PDAC: pancreatic ductal adenocarcinoma
PI3K: phosphatidylinositol 3-kinase
PKC: protein kinase C
PLOD2: procollagen-lysine, 2-oxoglutarate 5-dioxygenase 2
PTEN: phosphate and tension homology deleted on chromosome ten
RAC1: Ras-related C3 botulinum toxin substrate 1
SCC: squamous cell carcinoma
SMA: smooth muscle actin
STAT: signal transducers and activators of transcription
TACS: tumor-associated collagen signature
TGF-β: transforming growth factor-β
TNF: tumor necrosis factor
YAP: Yes-associated protein
